# Predictive modeling in case-control single-nucleotide polymorphism studies in the presence of population stratification: a case study using Genetic Analysis Workshop 16 Problem 1 dataset

**DOI:** 10.1186/1753-6561-3-s7-s60

**Published:** 2009-12-15

**Authors:** Niloofar Arshadi, Billy Chang, Rafal Kustra

**Affiliations:** 1 Dalla Lana School of Public Health, University of Toronto, Toronto, Ontario, M5T 3M7, Canada

## Abstract

In this paper, we apply the gradient-boosting machine predictive model to the rheumatoid arthritis data for predicting the case-control status. QQ-plot suggests severe population stratification. In univariate genome-wide association studies, a correction factor for ethnicity confounding can be derived. Here we propose a novel strategy to deal with population stratification in the context of multivariate predictive modeling. We address the problem by clustering the subjects on the axes of genetic variations, and building a predictive model separately in each cluster. This allows us to control ethnicity without explicitly including it in the model, which could marginalize the genetic signal we are trying to discover. Clustering not only leads to more similar ethnicity groups but also, as our results show, increases the accuracy of our model when compared to the non-clustered approach. The highest accuracy is achieved with the model adjusted for population stratification, when the genetic axes of variation are included among the set of predictors, although this may be misleading given the confounding effects.

## Background

Predictive modeling of case-control single-nucleotide polymorphism (SNP) data, using modern statistical and machine learning methods is a viable alternative to classical statistical genetic approaches that utilize one-marker-at-a-time hypothesis testing. With emphasis on prediction, a well executed modeling strategy can lead to a generalizable prognostic model that may form a basis for a genetic test. Some machine learning models, such as random forests [1] and gradient boosting machine (GBM) [2], can also rank covariates (here, SNPs) in terms of their predictive power. This presents a useful alternative to univariate testing because the promising SNPs are identified in the context of a multivariate predictive model that can discover multi-loci associations. Further, an unbiased estimate of predictive performance of such models also presents a useful summary of diagnostic value as opposed to a sea of *p*-values one obtains from association studies.

**Figure 1:**
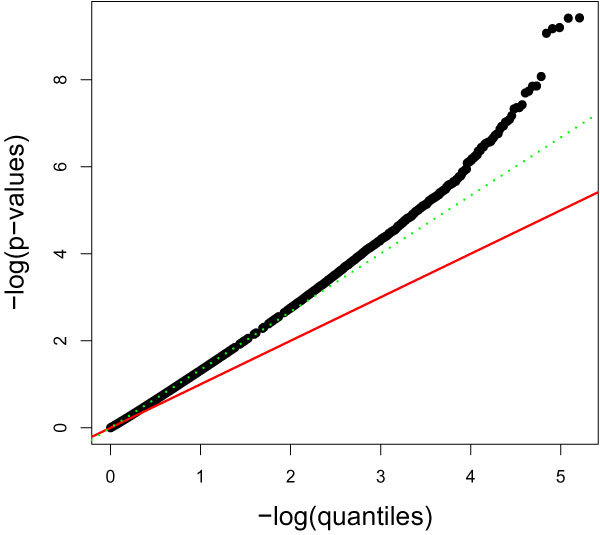
**QQ-plot for the original data (inflation factor = 1.335)**.

Genetic Analysis Workshop 16 Problem 1 dataset is a case-control study of rheumatoid arthritis (RA) with a total sample size of 2,062 cases and controls, each genotyped using Illumina HumanMap 500 k array interrogating 545,080 SNP markers. Classical analysis using a one degree of freedom trend test of association shows a significant *p*-value inflation with the slope of 1.33 (Figure 1), which indicates significant confounding is present. Also, *p*-values obtained by 100,000 permutations exhibit very similar pattern, suggesting that asymptotic chi-square test is not the cause. The *p*-value inflation is also reported in the original publication [3], and population stratification is suggested as a cause.

In this paper, we describe results when a dataset with potential ethnicity complexity is subjected to predictive analysis using GBM predictive model, and compare the obtained results to analysis based on original data with ethnicity complexity.

## Methods

We have set aside 33% of the data as a test set (285 cases and 400 controls) for the final assessment of predictive performance. The remainder of the data (1,370 subjects), training data, is split into five folds and is used to develop and optimize the GBM genetic predictive model for lifetime risk of developing RA. More precisely, during the parameter optimization process, the model is trained using four folds -- called CV-training folds -- and is evaluated on the fifth fold -- validation set.

### Data preprocessing

We first preprocessed the data for outlier detection, which resulted in seven controls (D0009324, D0009459, D0005318, D0011466, D0012257, D0006047, and D0012446) marked as outliers. We followed the general description of Plenge et al. [3], i.e., a subject-to-subject covariance matrix of a set of 120,000 minimally correlated SNPs is computed, and the four eigenvectors corresponding to the top four eigenvalues of the covariance matrix are extracted (a screeplot of eigenvalues was used to decide on the number of selected eigenvalues). Those subjects whose respective entry in at least one eigenvector differed from the mean by more than six standard deviations were removed from any downstream analysis, and the eigenvectors were recomputed.

Then the SNPs were preprocessed as follows: 1) SNPs with less than 1% minor allele frequency were removed; 2) SNPs with less than 5% missing values were replaced with major alleles, or were excluded from the analysis if missing values were more than 5%; 3) tag SNPs were selected with linkage disequilibrium (LD) greater than 0.8 as measured by R^2 ^in PLINK 1.03 using the following option: indep-pairwise 50 10 0.80; and 4) because we are particularly interested in discovering novel SNPs predicting the RA risk, we removed SNPs in 6p21 and 8p23 regions. However, we also present some results with these two regions included.

At the end of preprocessing step, we were left with 2,055 subjects and 351,660 SNPs per subject. This data was then divided into training and test sets, and cross-validation (CV) folds as described above.

### SNP selection and cross-validation

The aim of the SNP selection process is to find a subset of SNPs to include in the predictive model to maximize its predictive accuracy. The selected SNPs may be investigated further by bioinformatics methods or low-throughput assays.

The SNP subsets we considered were based on univariate *p*-value thresholds. We used *p*-value correction procedure described previously [4] and PLINK software 1.03 [5] to compute *p*-values when the four eigenvectors of the covariance matrix (as explained in the data preprocessing section) are included in the logistic regression model for a given SNP. Then, for various *p*-value thresholds a GBM model is built using the SNPs below the threshold. This is done repeatedly within a CV framework as follows. The four folds of the training set are used as a CV-training set. The *p*-values for the subjects in the four folds are re-computed at each iteration, and SNPs with *p*-values below the threshold are selected. Then, a clustered GBM model is built, and the value of area under curve (AUC) of the accuracy of predictive model is computed on the fifth fold -- validation set. The process is repeated five times, and we report the average AUC.

### Model correction for ancestry confounding

The population stratification present in the dataset, which seems to cause significant *p*-value inflation, can also be detrimental to building and validating a multivariate predictive model. To deal with that, we build separate predictive models on subsets of the data that are derived by clustering the subjects using the axis of genetic variation. Hence, each cluster will have a reduced overall genetic variability, which is usually assumed to be reducing the underlying ethnicity differences. Another way to see our procedure is to think of building predictive models conditional on certain ethnicity, *P*(*Y*|*X*, *T *= *t*). Here *Y *is an outcome variable, *X*, the genetic (SNP) vector, and *T *is a latent ethnicity value, which is assumed to be the same for all subjects in a cluster. Of course we only have crude approximations to underlying ethnicities so our conditioning on *T *= *t *is only an approximation. A more usual way would be to build a model that is adjusted for ethnicities by incorporating variable *T *directly into the model. However, as we demonstrate in the later section (with an "adjusted" GBM model), the axis of variation variables are strong predictors and can easily overshadow true genetic signal. Further, the predictive performance of such a model cannot be properly estimated using a dataset with population stratification present.

### Deriving axes of genetic variation

Here we present our approach to deriving the axis of variation which is equivalent to that presented by Price et al. [4], but allows us to calculate the eigenvectors of a test case based on the axis derived on the training set. This is necessary so that we can obtain unbiased estimates of prediction error.

In what follows, we denote X_train _to be the matrix where each row contains the SNPs for a subject in the training set. The test set matrix X_test _will be defined similarly. The zygosity of the SNPs is encoded as 0, 1, or 2, indicating the number of minor alleles. We standardize X_train _as suggested by Price et al. [4]. We also standardize X_test_, except that we use the column means and standard deviations from X_train _for column standardization, and subtract each row of X_test _using X_test_'s row means. We then perform singular value decomposition on X_train_:

We cluster the training subjects by performing partitioning around medoids (PAM) on the first four column vectors of U, which will provide us three medoids (m_i_, i = 1, 2, 3) representing the centroids of the three clusters in the four-dimensional space.

Next, given the SNP vector for a test subject (a row of X_test_) x_test_, we calculate:

where V_sub _contains the first four columns of V from Eq. (1) and D_sub_ is the 4x4 upper-left sub-matrix of D in Eq. (1). Then we assign x_test _to one of the three clusters from the training data using:

where ||.|| is the Euclidean norm. The above is equivalent to the EigenStrat procedure [4], which obtains the same matrix U as in Eq. (1). Using singular value decomposition allows us to project a test set observation onto the axes of genetic variation of the training data. Therefore, we can perform the clustering step of model building using only the training (or CV-training) set, and to subsequently predict the case status on an unseen data in the test or validation set.

Hence, all steps of our model building (SNP selection, clustering, and model training) are performed only on the training set. This allows us to obtain unbiased estimated of the predictive performance of our modeling strategy.

### Gradient boosting machine (GBM)

To model *P*_*t*_(*Y*|*X*, *T *= *t*), we use an ensemble of predictive models (GBM) that is based on the boosting paradigm [2]. The basic model unit in GBM is a small decision tree that allows us to model genetic interaction effects. Many such trees are combined in what is effectively a regularized logistic model with trees as individual covariates.

In our experiments, we utilize the 'gbm' package in R, where we set the interaction depth of each tree to 5, shrinkage factor to 0.01, bag fraction to 0.8, and the number of minimum subjects in each node to 10. These parameters values were picked empirically by examining the result of a small experiment involving various parameter settings, but were not formally optimized. The number of trees in a model should depend on a sample size and was simply set equal to the number of subjects in the training set, which varies among clusters. We also present the results with GBM applied to the whole dataset (unclustered) and applied to the whole dataset augmented with axis of variation variables (adjusted GBM) using the same parameter settings.

## Results

To decide on the number of clusters, we use the average Silhouette value [6] when clustering the training set into two, three, and four groups. Our results on the training set show that the average Silhouette value is higher for three and four groups, but because since there are as few as 93 subjects in a four-group split, we set the number of clusters to three.

**Figure 2:**
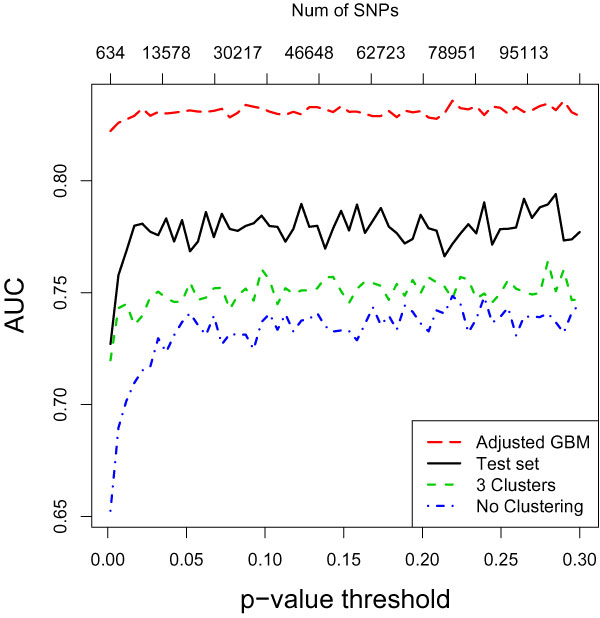
**Comparing AUC of GBM predictive models**. The average AUC based on 5-fold CV on the training set for "Adjusted GBM", "3 Clusters", and "No clustering".

We compare the accuracy of GBM using five-fold CV in the following three scenarios (Figure 2): 1) the average accuracy of GBM model with no clustering performed to solve the population stratification; 2) the average accuracy in the case in which four eigenvectors are used as ethnic covariates in the GBM model in addition to the *p*-value-filtered SNPs (an ethnicity adjusted GBM); and 3) the average accuracy of the clustered GBM on our CV-validation set. We also compare the accuracy of various GBM models evaluated on the test set.

In order to explore the top-ranked SNPs selected by GBM, we use the relative influence measure in the GBM package. There are 19,701 SNPs used in the GBM model with *p*-value = 0.05 (we chose this traditional threshold because around *p*-value = 0.05, the performance of the GBM classifiers seems to stabilize, Figure 2). Using the four eigenvectors V_1_, V_2_, V_3_, and V_4 _(as explained in the Methods section), we cluster the whole dataset into three clusters, C_1_, C_2_, C_3_, with 1370, 475, and 210 subjects, respectively. We compare the top 20 SNPs selected by the adjusted GBM, as well as top 20 SNPs chosen by GBM models in each of the three clusters in two scenarios: 1) 6p21 and 8p23 regions are excluded; 2) all SNPs that satisfy our quality control constraints and LD threshold (as explained in the Data preprocessing section) included.

In the first scenario, as expected, V_1_, V_2_, and V_4 _appear at the top of the list for the adjusted GBM (the *p*-values for these eigenvectors are less than 10^-24^), and there are five SNPs (including rs6596147 on chromosome 5) that appear in both C_1 _cluster and adjusted GBM model. Also, rs2476601, one of the SNPs in *PTPN22 *on chromosome 1 [3], appears in the top 20 list for the adjusted GBM and for the C_2 _cluster.

In the second scenario that includes SNPs from 6p21 and 8p23, we notice 11 common SNPs between C_1 _cluster and adjusted GBM, but 7 of these are in 6p21 region. That suggests that SNPs on the 6p21 region are strongly associated with RA and are chosen by both full-data adjusted GBM model and GBM from the largest cluster C_1_, perhaps overshadowing other SNPs with more moderate signal strengths. However, the top 20 list for C_3 _shows only two SNPs in 6p21 (one of which also appears in the adjusted and C_1 _and C_2 _models), but has 16 SNPs in common with the top 20 list for the C_3 _model that excludes SNPs from both 6p21 and 8p23 regions. That suggests that while the RA susceptibility of subjects in cluster C_1 _(and to a lesser extent in C_2_, which shares 5 SNPs in 6p21) is influenced by 6p21, genetic risk profile of subjects in C_3 _is much less dependent on that region. We also notice rs6596147 in 5q31.1 region appears again in the selected SNPs by adjusted and C_1 _GBM model, a finding that should be further explored given the mixed results reported in the literature on the association of 5q31 and the RA disease [7,8]. Also, rs2476601 in PTPN22 falls from the 8^th ^and 10^th ^to the 35^th ^and 169^th ^positions in the adjusted GBM and C_2_, respectively, suggesting that including SNPs on 6p21 may shadow signals of SNPs in other regions.

Comparing the top ranked SNPs from our models to their univariate *p*-values, we notice SNPs such as rs2395117 in 6p21, which has the smallest *p*-value = 1.6 × 10^-56 ^(that is in top 20 list of all models in the second scenario), and SNPs such as rs2691269 on chromosome 19 with *p*-value = 8.7 × 10^-3 ^(that is ranked 3,905 SNPs in the univariate *p*-value analysis), implying that top-ranked SNPs in GBM do not necessarily have the lowest *p*-values. It should be noted that there was no SNP in the top-ranked SNP list of any GBM model from the 8p23 region.

## Discussion

The main purpose of our five-fold CV was to select the *p*-value threshold that maximizes the accuracy of the prediction model on our training set. However, as Figure 2 shows, the GBM model is relatively robust with respect to the number of irrelevant SNPs included in the model. This is encouraging news, suggesting that such an expensive CV exercise may not be necessary with the GBM model.

We notice that the adjusted GBM outperforms all other predictors: this was expected because the four eigenvectors are highly correlated to the case status (their corresponding *p*-values were less than 10^-8^). However, such a model may not be able to discover novel SNPs with moderate signals because they may be overshadowed by strong eigenvector covariates. Also, its prediction error is likely underestimated: if deployed in the population with much different ethnicity distribution, such a model would likely fare much worse. This is because much of its predictive performance gains are likely due to predicting underlying ethnicity rather than disease status, which appears strongly confounded in this dataset.

Clustered GBM exhibits higher prediction accuracy compared to the unclustered GBM. Following the paradigm of "divide and conquer", that could be explained by the fact that clustering can make the classification problem easier by dividing the training data into more homogenous subject groups.

We also observe that we achieve a higher performance on the test set than the CV results. This is likely due to the 25% increase in the sample size for the model built for the test set prediction as compared with models built during CV.

## Conclusion

In this paper, we developed a prediction model of RA risk using a GBM predictive framework. To avoid confounding by ethnicity, the data were first clustered on the axes of genetic variations. An ensemble of three GBM classifiers were then trained on the three resulting clusters, which are assumed to be ethnically more homogenous. For predicting the case-control status of a subject in the test set, we first determined the cluster that the subject belonged to by projecting on the axis of genetic variation, and then applied the corresponding GBM model.

We are interested in further investigating the set of SNPs ranked higher by the clustered GBM model, and in comparing them with those reported in the literature. It would also be interesting to validate our model on another RA dataset with a different ethnicity distribution to confirm that we have, at least partially, avoided confounding when developing our prediction model.

## List of abbreviations used

AUC: Area under curve; CV: Cross-validation; GBM: Gradient boosting machine; LD: Linkage disequilibrium; RA: Rheumatoid arthritis; SNP: Single-nucleotide polymorphism.

## Competing interests

The authors declare that they have no competing interests.

## Authors' contributions

NA preprocessed the data, performed SNP selection, and cross-validated experiments. BC carried out the clustering and classification experiments. RK conceived of the study, and participated in its design and coordination.
